# Contribution of Eukaryotic-Type Serine/Threonine Kinase to Stress Response and Virulence of *Streptococcus suis*

**DOI:** 10.1371/journal.pone.0091971

**Published:** 2014-03-17

**Authors:** Haodan Zhu, Junming Zhou, Yanxiu Ni, Zhengyu Yu, Aihua Mao, Yiyi Hu, Wei Wang, Xuehan Zhang, Libin Wen, Bin Li, Xiaomin Wang, Yang Yu, Lixin Lv, Rongli Guo, Chengping Lu, Kongwang He

**Affiliations:** 1 Institute of Veterinary Medicine, Jiangsu Academy of Agricultural Sciences, Key Laboratory of Veterinary Biological Engineering and Technology of Ministry of Agriculture, National Center for Engineering Research of Veterinary Bio-products, Nanjing, China; 2 Jiangsu Co-innovation Center for Prevention and Control of Important Animal Infectious Diseases and Zoonoses, Yangzhou, China; 3 Key Lab of Animal Bacteriology, Ministry of Agriculture, Nanjing Agricultural University, Nanjing, China; University of South Dakota, United States of America

## Abstract

*Streptococcus suis* serotype 2 (SS2) is an important swine and human pathogen responsible for septicemia and meningitis. The bacterial homologues of eukaryotic-type serine/threonine kinases (ESTKs) have been reported to play critical roles in various cellular processes. To investigate the role of STK in SS2, an isogenic *stk* mutant strain (Δ*stk*) and a complemented strain (CΔ*stk*) were constructed. The Δ*stk* showed a significant decrease in adherence to HEp-2 cells, compared with the wild-type strain, and a reduced survival ratio in whole blood. In addition, the Δ*stk* exhibited a notable reduced tolerance of environmental stresses including high temperature, acidic pH, oxidative stress, and high osmolarity. More importantly, the Δ*stk* was attenuated in both the CD1 mouse and piglet models of infection. The results of quantitative reverse transcription-PCR (qRT-PCR) analysis indicated that the expressions of a few genes involving in adherence, stress response and virulence were clearly decreased in the Δ*stk* mutant strain. Our data suggest that SsSTK is required for virulence and stress response in SS2.

## Introduction

Signal transduction through reversible protein phosphorylation is a key regulatory mechanism of both prokaryotes and eukaryotes [1]. In prokaryotes, signal transduction is thought to be primarily conducted by two-component systems(TCS), consisting of histidine kinase sensors and their associated response regulators [2]. Eukaryotic-type serine/threonine kinases (ESTKs) and cognate phosphatases (ESTPs) operate in many bacteria [3]–[12], constituting a signaling network independent of the canonical TCS circuits. Prokaryotic ESTKs have been shown to regulate various cellular functions, which include cell growth and division [13], metabolism [1], [14], stress response [15]and adaptation to changes in environmental conditions [16]–[18]. STKs also play a role in virulence of some bacterial pathogens such as *Streptococci* [5]–[8], *Mycobacterium tuberculosis* [19], [20],*Yersinia pseudotuberculosis* [21] and *Staphylococcus aureus* [22].

*Streptococcus suis* is a major swine pathogen responsible for a wide range of diseases, including septicaemia, meningitis, endocarditis, arthritis, and even acute death [23]. *S. suis* is also an important zoonotic agent afflicting people in close contact with infected pigs or pork-derived products. Thirty-three serotypes (types 1–31, 33, and 1/2) have been described based on capsular polysaccharides [24]. Serotype 2 (SS2) is the most virulent and most frequently isolated serotype. To date, many *S. suis* virulence factors have been identified, including capsular polysaccharide (CPS) [25], [26], opacity factor (OFS) [27], hemolysin (suilysin) [28], fibronectin- and fibrinogen-binding protein (FBPS) [29], Inosine 5-monophosphate dehydrogenase (IMPDH) [30], autolysis [31] and some regulators such as TCS *Sal*K/R [32], CiaR/H [33], orphan regulator *Cov*R [34], *Rev*S [35] and others [36].

The major ecological niche harbored by *S. suis* is the epithelium of the upper respiratory tract in pigs. Critical events in the development of disease are bacterial invasion from the mucosal surface into deeper tissues and the blood circulation, survival in blood, and invasion from blood to various host organs [37]. The ESTKs have been implicated in various steps of bacterial pathogenesis, as shown in *Streptococcus pyogenes*, SP-STK mutants exhibited decreased adherence to human pharyngeal cells [5].In *Streptococcus agalactiae*, both theΔ*stk1* andΔ*stp1*Δ*stk1* mutants are significantly impaired for survival in whole blood [38]. In *Streptococcus pneumoniae*, StkP can promote persistence of bacterial *in vivo* and contribute to survival in various stress environments [7], [15]. The signaling molecules ESTK and ESTP are well characterized in some other *Streptococci* [5]–[8]. However, it is not known whether the pathogenicity of *S. suis* requires a similar STK/STP system.

The genome analysis has revealed the presence of homologues of ESTK and ESTP in *S. suis* genome, which have been designed as SsSTK and SsSTP, respectively. The SsSTP was identified by SSH in *S suis* strain and involved in pathogenesis of the bacteria [39]. But the role of SsSTK has not been thoroughly elucidated in *S. suis* infection. In the present study, we constructed a mutant strain(Δ*stk*) as well as a complemented strain(*C*Δ*stk*), and evaluated their virulence *in vitro* and *in vivo*, which helped us to understand the precise role of the ESTK gene in the pathogenicity of *S. suis.*

## Materials and Methods

### Ethics statement

All animals used in this study, and animal experiments, were approved by Department of Science and Technology of Jiangsu Province. The license number was SYXK(SU) 2010–0005. All efforts were made to minimize suffering.

### Bacterial strains, plasmids, and growth conditions

Bacterial strains and plasmids used in this study are listed in Table 1. Strain SS2-1was isolated from a dead pig with septicaemia in Jiangsu province in 1998 and has been confirmed as virulent on the basis of animal experiments. SS2 strains were grown in Todd-Hewitt broth (THB) (Difco Laboratories, Detroit, MI) medium or plated on THB agar containing 5% (vol/vol) sheep blood. *Escherichia coli* strains were cultured in Luria broth (LB) liquid medium or plated on LB agar. SS2 strains were grown in THB supplemented with 2% yeast extract (THY) for preparation of competent cells. Antibiotics (Sigma) were supplemented to culture media as required, at the following concentrations: spectinomycin (Sp), 100 μg/mL for *S. suis*, and 50 μg/mL for *E. coli*; chloramphenicol (Cm), 4 μg/mL for *S. suis*, and 8 μg/mL for *E. coli*.

**Table 1:** Bacterial strains and plasmids used in this study.

*Bacterial strains*	*Description*	*Source or reference*
SS2-1	Serotype 2, clinical isolated virulent strain, *mrp* ^+^ *ef* ^+^ *sly* ^+^	Our laboratory
Δ*stk*	The deletion mutant of stk with background of SS2-1	This work
CΔ*stk*	Complemented strain of Δ*stk*; Spc^R^, Cm^R^	This work
*E. coli* TOP10	Cloning host for maintaining the recombinant plasmids	Tiangen
Plasmids		
pMD18-T	Clone vector	Takara
pSET4s	*E. coli–S*. suis shuttle vector; replication function of pG+host3 and pUC19, lacZ' Spc^R^	Takamatsu (2001)
pSET4sΔ*stk*	A recombinant vector with the background of pSET4s, designed for knockout of *stk*	This work
pSET2	*E. coli-S*. suis shuttle vector; Spc^R^	Takamatsu (2001)
pSET2-STK	pSET2 containing the intact STK gene and promoter, Spc^R^	This work
pR326	*E. coli* plasmid, Ap^R^, Cm^R^	Claverys *et al*. (1995)

### Co-transcription assay

**Figure 1:**
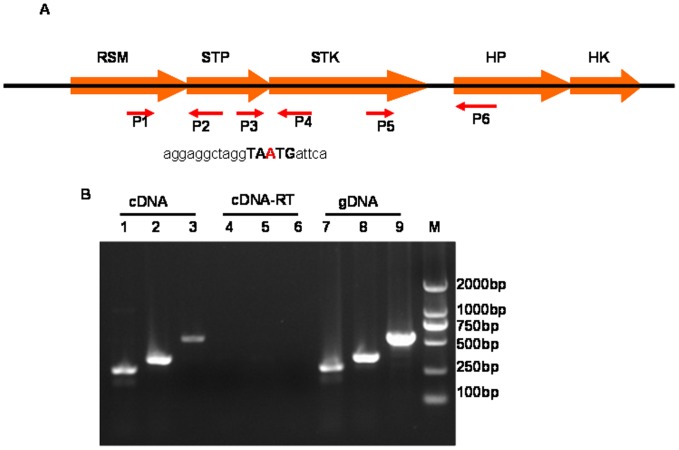
The genomic context of *stk* and *stp* in *S. suis*. A. Schematic of the *stk/stp* genetic locus showing primer annealing sites. The one nucleotide by which the two genes overlap are in red font. B. Co-transcription analysis of the four genes RSM to HP using reverse transcription (RT-) PCR analysis with cDNA, cDNA-RT(cDNA reaction mixtures without reverse transcriptase) or genomic DNA (gDNA) as templates. Lanes 1, 4, and 7 represent the amplification using primer set P1 and P2, lanes 2, 5, and 8 represent the amplification using primer set P3 and P4, lanes 3, 6, and 9 represent the amplification using primer set P5 and P6.

Total RNA was extracted from *in vitro* late logarithmic phase (OD 600, 0.6–0.8) bacterial culture using the EZNA bacterial RNA kit (Omega, USA) according to the manufacturer's protocol. cDNAs were reverse transcribed using a PrimeScript RT-PCR kit (Takara, Dalian, China). An identical reaction was performed without reverse transcriptase as a negative control**.** cDNA with or without reverse transcriptase and genomic DNA (gDNA) were used as templates in PCRs using specific primer sets specific for overlapping (P3/P4), and outermost regions of *stp* and *stk* (P1/P2 and P5/P6), as shown in Fig. 1A

### Construction of an isogenic *stk* deletion mutant and complemented strains

Construction of Δ*stk* -knockout mutant: the *stk* deletion mutant was performed as a previously described procedure [40]. *stk* was inactivated by allelic exchange with a chloromycetin resistance (Cm^R^) cassette [40]. Briefly, DNA fragments were amplified from the gDNA of SS2-1 by PCR with two pairs of specific primers, L1/L2 and R1/R2, carrying *Hind*III/*Sal* I and *BamH* I/*EcoR* I restriction enzyme sites, respectively (Table 2). Fragments were digested with the corresponding restriction enzymes and directionally cloned into a temperature-sensitive *S. suis-E. coli* shuttle vector pSET4s [40]. The *cat* gene cassette (from pR326 [41]) was inserted at the *Sal* I/*BamH* I sites to generate the *stk*-knockout vector pSET4sΔ*stk.*To obtain the isogenic mutant Δ*stk*, the SS2-1 competent cells were electrotransformed with pSET4sΔ*stk* as described previously [40]. For all of the Cm^R^ transformants, a colony PCR with primers IN1/IN2 was performed to detect the presence of *stk* in the genome. Candidate mutants in which the *stk* gene failed to be amplified were further verified by PCR assays with primers CAT1/CAT2, OUT1/CAT2 and CAT1/OUT2, which was confirmed by DNA sequencing.

**Table 2:** Oligonucleotide primers used in this study.

*Primers*	*Primers sequence (5′–3′)* a	*Functions*
P1	ACAGGTGACACTAGAACACCCTCAA	For co-transcription assay
P2	AGCAATATGGCCGGCACGGT	For co-transcription assay
P3	TGCTTCACATTACGGAGGAGGCT	For co-transcription assay
P4	TCGCACCGCAACATCGTTGGA	For co-transcription assay
P5	GCATCAACCAGAACCAGTAGCGA	For co-transcription assay
P6	GGTCCGACTGGCCTCATTGGC	For co-transcription assay
L1	ACCCAAGCTTTGCTAAGGAAAACCAAGAGG	Upstream border of *stk*
L2	ACGCGTCGACTACCTAGCCTCCTCCGTA	
R1	GCGCGGATCCAAGATGAATAAGGTTGGGG	Downstream border of *stk*
R2	ACCGGAATTCCACCCAGGAAACTTACTCG	
CAT1	ACGCGTCGACCACCGAACTAGAGCTTGATG	Cat^R^ gene cassette
CAT2	GCGCGGATCCTAATTCGATGGGTTCCGAGG	
IN1	AGGGTTGAACTAGAAGGG	An internal fragment of stk
IN2	CTGTCGCTTCTTCTGTGA	
OUT1	CAAAGGTCTGGACGCCAG	For PCR assay
OUT2	ATCGGGACTATTGACCGCT	
CPS2J-1	CAAACGCAAGGAATTACGGTATC	For PCR assay
CPS2J-2	GAGTATCTAAAGAATGCCTATTG	
Pim-1	ACATGCATGC ATGGAGGCAGGACAGGTAT	For amplification promoter of IMPDH
Pim-2	GCGCGGATCCGTTCTTTCCTTTCTTTTGGG	
STK-F	GCGCGGATCCATGATTCAAATCGGTAAGATCTT	For amplification SsSTK ORF gene
STK-R	GGTGAATTCTTATTGTCCGCTACCTGTTG	
16SrRNA-1	GTTGCGAACGGGTGAGTAA	For Real-time PCR
16SrRNA-2	TCTCAGGTCGGCTATGTATCG	For Real-time PCR
GAPDH-1	CTTGGTAATCCCAGAATTGAACGG	For Real-time PCR
GAPDH-2	TCATAGCAGCGTTTACTTCTTCAGC	For Real-time PCR
FBPS-1	GGTGGCCCAGCAGGCCAATG	For Real-time PCR
FBPS-2	CCGCCAATCCCTGCTCCTGC	For Real-time PCR
MRP-1	GTTGAGCAAGTTGAAGCGCA	For Real-time PCR
MRP-2	GGTACCTTCGCCATCACCAA	For Real-time PCR
EF-1	AGGCTGCTAAGGATGCCGTTGC	For Real-time PCR
EF-2	CGCCTACTGCTTCTGCACTGTCC	For Real-time PCR
IMPDH-1	TCGACCAACATGACAAGCGA	For Real-time PCR
IMPDH-2	ATCCTTCGCAGCATTTGGGA	For Real-time PCR
SsnA-1	TGCCTTTGCTCAAGCTCTTCGTG	For Real-time PCR
SsnA -2	TGCCTTTTTAGTTGCCCGGCCA	For Real-time PCR
SspA-1	TGACCAGGCAGTTGAAGCAGCG	For Real-time PCR
SspA-2	TGCCTGAGCGCTTGTCAGAACG	For Real-time PCR
Sly-1	TGATGAACCAGAATCTCCAAGCAAG	For Real-time PCR
Sly-2	GTCTTGATACTCAGCATTGCCACTA	For Real-time PCR
SodA-1	GTAAGAAACAATGACCCTTCACCAC	For Real-time PCR
SodA-2	GCAAAGCAATTCCCAGAAAAGAGCA	For Real-time PCR
Ad-1	TGCCTTTGCTCAAGCTCTTCGTG	For Real-time PCR
Ad-2	TGCCTTTTTAGTTGCCCGGCCA	For Real-time PCR
OppuABC-1	CAGAGTCGCCGTTCCGATAA	For Real-time PCR
OppuABC-2	GGAACCTTGCCAGCAGTAGT	For Real-time PCR

a Underlined nucleotides denote enzyme restriction sites.

As the sequence and location of the endogenous promoter that facilitates *stk* transcription in *S. suis* are unknown, we used the promoter sequence of the IMPDH [30]for the construction of a genetic complementation plasmid. This fragment was amplified from SS2-1 gDNA by PCR using the primers P*im*-1/P*im*-2, and cloned into *Sph*I and *BamH*I sites of *Sph*I/*BamH*I-digested *E. coli-Streptococcus* shuttle vector pSET2 [41]. The gene encoding SsSTK was amplified by PCR using the primers STK-F/STK-R and cloned downstream of the P*im* promoter in pSET2 at the *BamH*I*/Eco*RI sites to generate the *stk* -complementing plasmid pSET2-STK. This recombinant vector was electrotransformed directly into the Δ*stk* to obtain the complemented strain *C*Δ*stk* using previously reported method [40].

### *In vitro* stress experiments

#### Temperature stress

To compare the effect of high temperature stress on the wild-type strain and its derivatives, the different strains were grown in THY broth to the mid-exponential phase. Aliquots of the appropriate size were diluted to an OD600 of 0.1 with approximately 10 mL of fresh THY broth. All of the cultures were incubated for 12 h at 37°C and 40°C in 5% CO_2_. Subsequently, the number of colony forming units (CFU) per millimeter was determined by dilution plating on THY agar plates. All experiments were conducted in duplicate and repeated three times.

#### Acid tolerance

To study the role of SsSTK in acid tolerance, the different *S. suis* strains were grown in THY broth that had been adjusted to pH 7.0 with HCl. Cultures were harvested at mid-exponential phase by centrifugation at 4000 g at 4°C for 10 min, washed once with 0.1 M glycine buffer (pH 7.0), and then subjected to acid killing by incubating the cells for 45 min in THY of different pH from 4.5 to 7.0 adjusted by HCl. Surviving cells were appropriately diluted and plated on THY agar plates. All experiments were conducted in duplicate and repeated three times. The means of three experimental trials were used to characterize the survival ratio of the different strains.

#### Oxidative stress

To evaluate oxidative stress tolerance, different *S. suis* strains were challenged with H_2_O_2_.The sensitivity of cells to H_2_O_2_ was tested by exposing aliquots of cultures (10^7^ CFU/mL; OD600 0.6) grown in THY broth at 37°C to 40 mM and 80 mM H_2_O_2_ for 20 min. Viable cells were counted by plating them onto THY agar plates before and after exposure to H_2_O_2_, and results were expressed as percentages of survival. All experiments were conducted in duplicate and repeated three times.

#### Osmotic stress

Adaptability of the wild-type strain and its derivatives to osmotic stress was evaluated by monitoring bacterial growth in THY broth containing 400 mM NaCl. The overnight cultures of SS2-1, Δ*stk* and the *C*Δ*stk* were diluted in fresh THY broth with and without NaCl to obtain at OD600 of 0.2. Samples were inoculated at 37°C for 8 h. At 1 h intervals, bacterial growth was monitored by measuring the OD600. All experiments were repeated three times.

### Bacterial adherence assay

Adherence assays were performed as previously described [42] with several modifications. The human laryngeal cell line HEp-2 was cultured in RPMI 1640 media (Invitrogen, USA), supplemented with 10% fetal calf serum (FCS), and maintained at 37°C with 5% CO_2_. Log phase bacteria were pelleted, washed twice with PBS (pH 7.4), and resuspended in fresh cell culture medium without antibiotics at an appropriate density (1×10^7^ CFU/mL). Confluent monolayers of HEp-2 grown in 24-well plates were infected with 1 mL aliquots of a bacterial suspension. After incubation for 90 min at 37°C, the monolayers were washed three times with PBS, digested with 100 μL of 0.25% trypsin–0.1% EDTA, and then lysed by the addition of of 900 μL 0.025% Triton X-100 following repeated pipetting to release all bacteria. Serial dilutions of the cell lysate were plated onto THY agar to enumerate viable bacteria. All experiments were conducted in triplicate and repeated three times. The means of three experimental trials were used to characterize the adherence capacity of the different strains.

### Survival in the presence of swine whole blood

Blood samples were collected by venous puncture from high-health-status pigs that were considered to be free of SS2 as determined by an enzyme-linked immunosorbent assay [43]. Susceptibility assays were performed as previously described [44]. The SS2-1, Δ*stk* and *C*Δ*stk* were cultured to the early stationary growth phase. The cells were pelleted, suspended in RPMI 1640 medium to an OD 600 of 0.1, before diluting 1∶100 in RPMI 1640 medium. One mL of swine whole blood was mixed with 300 μL pig anti-SS2 serum and 100 μL of SS2 cells. Anti-SS2 serum was prepared in pigs by injecting whole bacterial cells as previously described [26]. Mixtures were incubated for 2 h at 37°C with occasional gentle shaking. Infected whole blood cultures were harvested at 0 h and 2 h. to determine bacterial survival using the plate count method. The first time point (0 h) was considered as the 100% viability control. All experiments were conducted in triplicate and repeated twice.

### Experimental infections of mice and pigs

#### Determination of Δ*stk* virulence in a CD1 mouse model

The CD1 mouse is an excellent model for *S. suis* infections [45]. A total of 155 female 6-week-old CD1 mice (Beijing Vital River Laboratory Animal Co., Ltd.; colonies derived from Charles River Laboratories) were used to assess virulence. 150 mice were randomly classified into 15 groups with 10 mice per group, and the other 5 mice were used as control. The log phase cultures of SS2 strains were centrifuged, the cell pellets were washed twice in PBS, and then suspended in THY. For each strain, five groups experimental mice were injected intraperitoneally(ip.) with 1.0 mL of a suspension of different strains at the following concentrations: 3.2×10^6^ CFU/mL, 1.6×10^7^ CFU/mL,8.0×10^7^ CFU/mL,4.0×10^8^ CFU/mL and 2.0×10^9^ CFU/mL. Five control mice were inoculated only with the medium solution (THY). Mortality was monitored until 7 days post-infection(PI) and we calculated the 50% lethal dose (LD_50_) value using the method of Reed and Muench [46].

#### Determination of viable bacteria in organs

Ten CD1 mice were assigned to two groups and used to assess the presence of viable bacteria in infected organs. Group 1 was inoculated by ip injection of 0.5 mL of a SS2-1 suspension (2.0×10^8^ CFU/mL), while group 2 received the same dose of the Δ*stk*, using the same inoculation route. Two control mice were inoculated with only the culture medium solution (THY). Blood samples were collected from the tail vein at 24 h PI, and at the same time all mice were euthanased. Bacterial colonization of the liver, spleen, kidney, and brain was also evaluated. Small samples of these organs weighing 0.2 g were trimmed, placed in 2 mL of PBS, and homogenized. Then we prepared dilutions of 100 μL of each homogenate in PBS, from 10^−2^ to 10^−5^, and plated the suspensions onto THY agar. Blood samples (100 μL) were also plated. Colonies were counted and expressed as CFU/g, for organ samples, and CFU/mL, for blood samples.

#### Experimental infection of piglets

A total of 20 high-health-status piglets (ages 4–5 weeks) which tested negative for SS2 by ELISA were used. To minimize the number of piglets used for the experiment, the virulence of SS2-1, Δ*stk* and *C*Δ*stk* was compared by inoculating with an equal dose (5×10^6^ CFU/piglet) instead of by determining their LD_50_. 18 piglets were randomly divided into 3 groups and were intravenously inoculated with either SS2-1, Δ*stk*, or CΔ*stk* (5×10^6^ CFU/piglet), respectively. Two control piglets were inoculated with PBS. Clinical signs and survival time were then recorded during the trial.

### Quantitative real time polymerase chain reaction (RT-PCR)

Bacteria samples collection, RNA extraction and cDNAs preparation were performed as described in Co-transcription assay. The two-step relative qRT-PCR was performed to analyze the expression profile of the virulence factors using SYBR Premix Ex Taq kit (TaKaRa, Dalian, China). The ABI 7500 RT-PCR system was used for relative qRT-PCR. The gene-specific primers used for the qRT-PCR assays were listed in Table 2. The housekeeping gene (16S rRNA gene) as the internal control was also amplified under the same conditions to normalize reactions. Reactions were carried out in triplicate. Dissociation analysis of amplification products was performed at the end of each PCR to confirm that only one PCR product was amplified and detected. The relative fold change after stimulation was calculated based on the 2^−ΔΔCt^ method [47].

### Statistical analyses

All the statistical analysis was performed on GraphPad Prism 5. One-way analysis of variance (ANOVA) was used to analyze the oxidative stress, bacterial adherence and survival in whole blood assays. Two-way ANOVA was performed on the stress experiments (temperature, acid and osmotic pressure) and qRT-PCR results. Mann-Whitney test was used to analyze the bacterial load in all organs examined. P<0.05 was considered significant. P<0.01 was considered highly statistical significant.

## Results

### Presence of *stk* in *S. suis* genome

Eukaryotic-type STK and STP (ESTK and ESTP) have been identified in a wide range of prokaryotes. An ESTP was identified by SSH in *S suis* strain and involved in pathogenesis of the bacterial in previous study [39].Genome analysis of SS2-1also revealed the presence of putative homologues of ESTP and ESTK, which encode a putative 738-bp, 246-amino-acid ESTP (possessing protein phosphatase 2C-specific motifs I to XI [48]) and a 1,995-bp, 665-amino-acid ESTK (possessing a complete set of STK-specific Hanks motifs I to XI [49]), respectively. The two genes are predicted to be co-transcribed based on one overlapping nucleotide (TA**A**TG) at the junction of their 3′and 5′ends (Fig. 1A), which we confirmed by reverse transcription (RT-) PCR analysis using primers P3 and P4, one of which hybridizes with a sequence within the *stk* and the other with one within the *stp* gene. RT-PCR analysis also demonstrated co-transcription of *stp* and *stk* with the adjacent up- and downstream genes, encoding a ribosomal RNA small subunit methyltransferase (RSM) and a hypothetical protein (HP), respectively (Fig.1B). The SsSTK (AGM49306) exhibits 60% and 52% amino acid sequence identity with the serine/threonine kinase of *Streptococcus sanguinis* SK353 and *Streptococcus pnuemoniae* D39, respectively.

### Characterization of the mutant strain Δ*stk*

**Figure 2:**
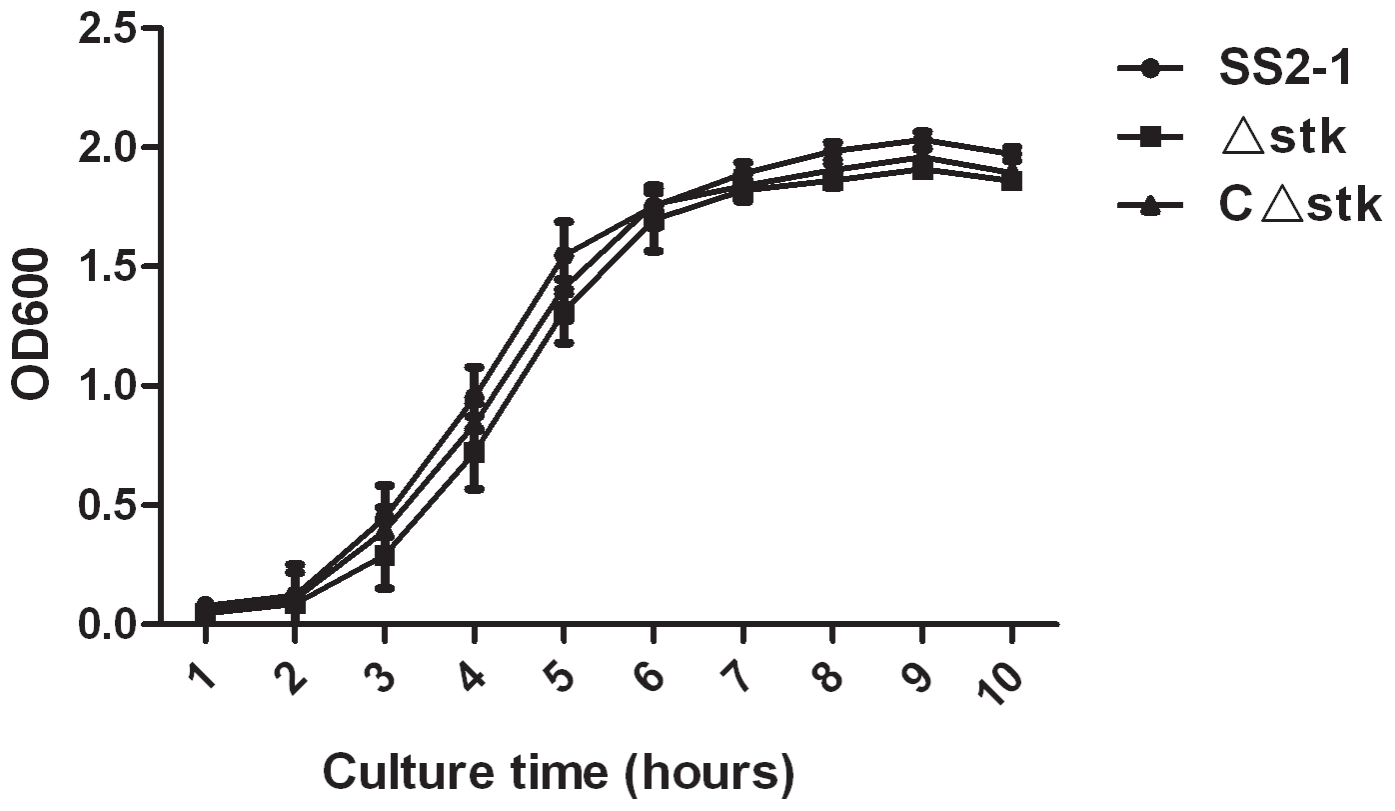
Growth characteristics of SS2-1, Δ*stk* and *C*Δ*stk*. Bacteria cell density is measured spectrometrically at 600

**Figure 3:**
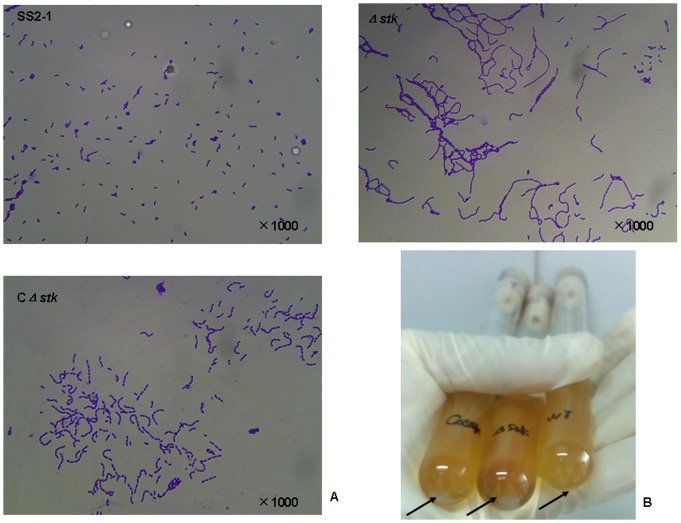
Cell morphology of SS2-1,Δ*stk* and CΔ*stk*. (A)Light microscope morphology of SS2 strains using Gram staining. (B)Sedimentation of bacteria cultured in THY for 12 h with gentle shaking. The arrows indicate the sedimented cells at the bottom of tubes.

**Figure 4:**
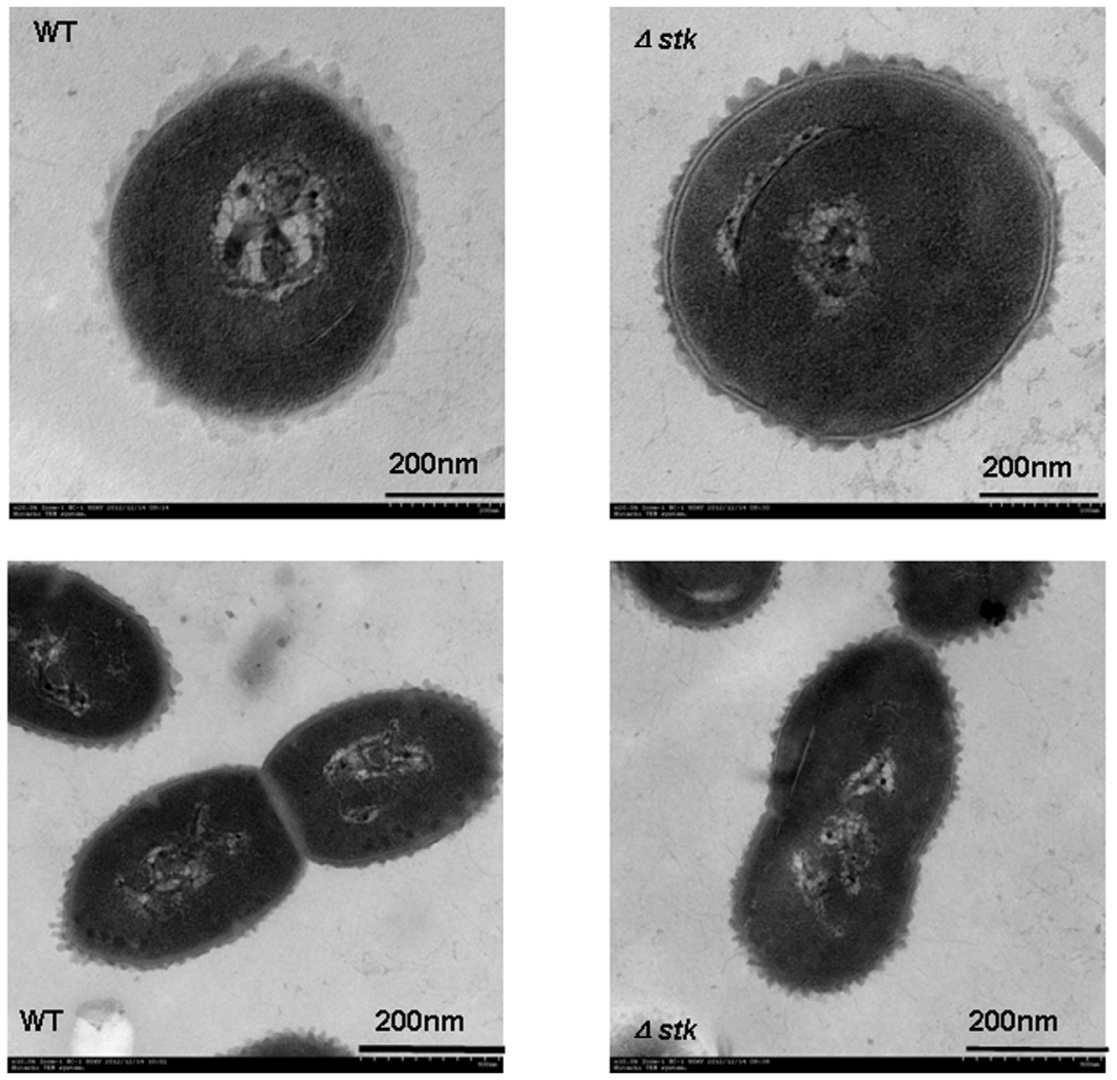
Transmission electron microscopy of SS2-1 and Δ*stk*. The bar indicates the magnification size.

Before studying the effect of *stk* inactivation on virulence of SS2 *in vivo*, we initially examined the growth characteristics of the null mutants *in vitro*. The OD600 of cultures of SS2-1, Δ*stk* and *C*Δ*stk* strains in THY broth at 37°C were determined. As shown in Fig 2, the growth of Δ*stk* and *C*Δ*stk* was slightly slower than the wild-type strain SS2-1. Moreover, the mean chain length of the Δ*stk* was found to be much longer than that of the wild-type strain under the same growth conditions (Fig 3A). The Δ*stk* cells have a tendency to settle during growth overnight in laboratory medium with gentle shaking, while the broth culture of wild-type strain cells was turbid, with fewer sedimented cells(Fig 3B).TEM revealed that the Δ*stk* displayed a noticeable increase in overall cell size(cell diameter,740±60 nm),compared with the wild-type strain (cell diameter, 510±50 nm)(Fig 4).These phenomena in the complemented strain *C*Δ*stk* were restored.

### SsSTK deficiency diminishes stresses tolerance of SS2

**Figure 5:**
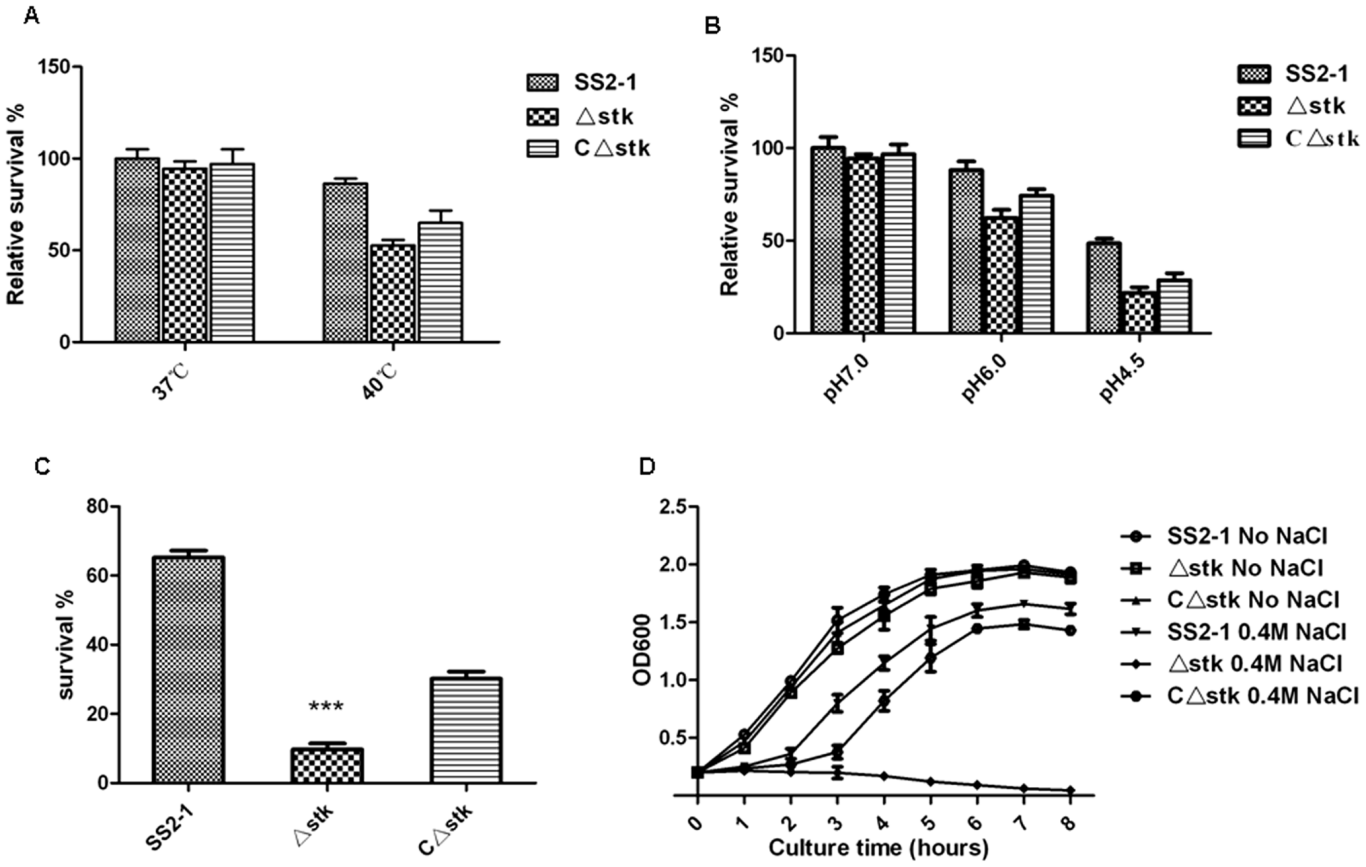
*In vitro* stress experiments. (**A**) **Thermal stress assay.** Bacteria were grown in THY broth to the mid-exponential phase. Aliquots of the appropriate size were diluted to an OD600 of 0.1 with approximately 10 mL of fresh THY broth. All of the cultures were incubated for 12 h at 37°C and 40°C, the number of colony forming units (CFU) per millimeter was determined. (**B**) **Acid tolerance assay.** Cultures were harvested at mid-exponential phase by centrifugation at 4000 g at 4°C for 10 min, washed once with 0.1 M glycine buffer (pH 7.0), and then subjected to acid killing by incubating the cells for 45 min in THY of different pHs. Surviving cells were appropriately diluted and plated on THY agar plates. (**C**) **Oxidative stress assay.** Bacterial cells were grown in THY medium at 37°C to an OD600 of 0.6 and the aliquots (10^7^ CFU) were used in each assay. Cells were incubated at 37°C for 20 min without or with 40 mM H_2_O_2_, and viable counts were carried out. Experiments were performed in duplicate and repeated three times. (**D**) **Osmotic stress assay.** Growth curves as measured by the ODs of the SS2-1, Δ*stk* and CΔ*stk* grown in THY and THY plus 400 mM NaCl. The growth of cultures was monitored from an initial OD600 of 0.2. Data are representative of three independent experiments.

During the infection process, *S. suis* is exposed to various stress factors, including nutritional deprivation, temperature shift, pH changes, increased osmolality, and reactive oxygen species generated by host phagocytes [50]. So we compared the growth characteristics of SS2-1, Δ*stk* and CΔ*stk* strains under different stress conditions *in vitro*. In contrast to the wild-type strain, which was unaffected at 40°C, the growth of the Δ*stk* was more susceptible to high temperature. The survival rate of the Δ*stk* and CΔ*stk* strains decrease sharply with the temperature increasing above 37°C(Fig 5A). The Δ*stk* showed reduced growth on low pH condition and less tolerance to an acid pH(Fig 5B). Decreased survival of the Δ*stk*, compared to that of the wild-type strain, was observed at 40 mM H_2_O_2_. After 20 min of treatment with 40 mM H_2_O_2_, 90% of the Δ*stk* cells were killed and 70% CΔ*stk* cells were killed, whereas 65% of the wild-type strain cells survived (Fig 5C). All the wild-type strain and its derivatives were completely killed exposed to 80 mM H_2_O_2_ over a 20 min period. Growth of the Δ*stk* in THY broth containing 400 mM NaCl was inhibited, where that of its wild-type strain and the CΔ*stk* were not (Fig 5D). These results suggest that the expression of SsSTK contributes to the resistance of *S. suis* to environmental stresses.

### Contribution of *Ss*STK to *in vitro* adhesion

**Figure 6:**
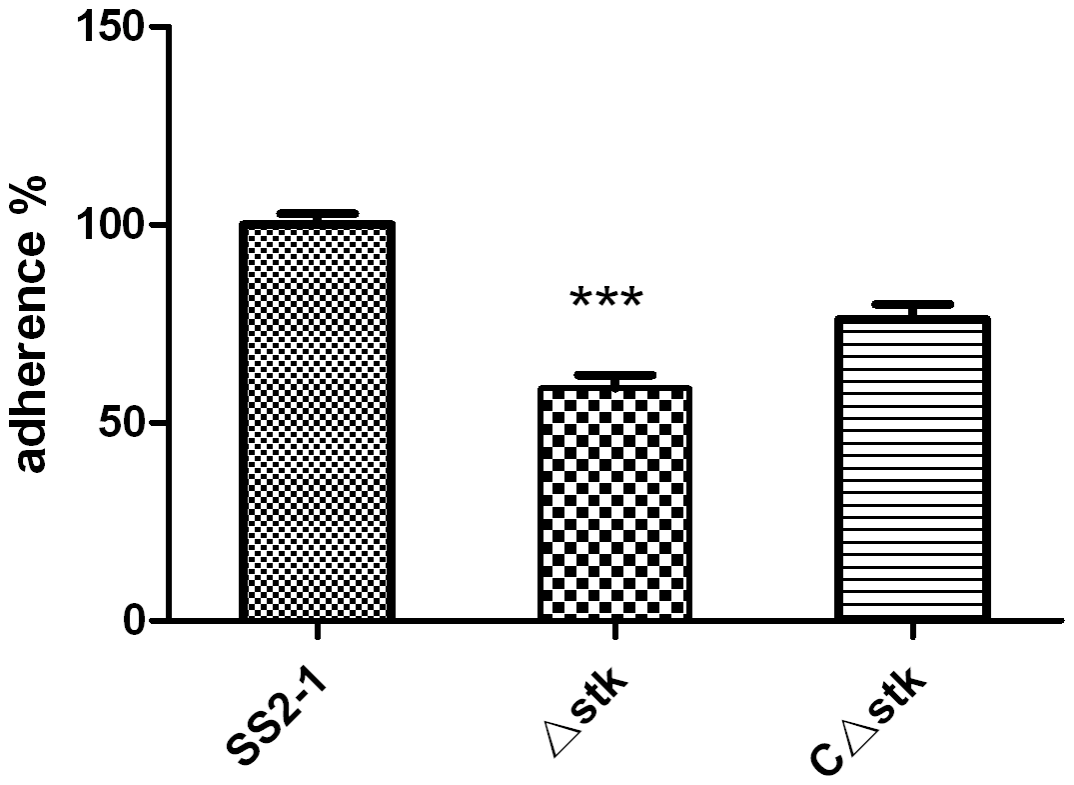
Effects of *stk* on SS2 adherence to HEp-2 cells. The mutant strain Δ*stk* showed significant reduced levels of adherence to HEp-2 cells compared to the adherencity of the parental strain SS2-1 (***p<0.001).

Adherence of pathogenic bacteria to the mucosal surface is considered to be an essential step in the infectious process. To determine whether the lack of SsSTK affected the cellular adhesion of SS2, the adherence efficiencies of the wild-type strain and its derivatives to HEp-2 cells were compared. As shown in Fig.6,there was a reduction of 41.3% in the adherence of the Δ*stk* compared with the SS2-1 and the adherence of *C*Δ*stk* was 75.9% of wild-type strain (***p<0.001).

### Susceptibility of whole blood

**Figure 7:**
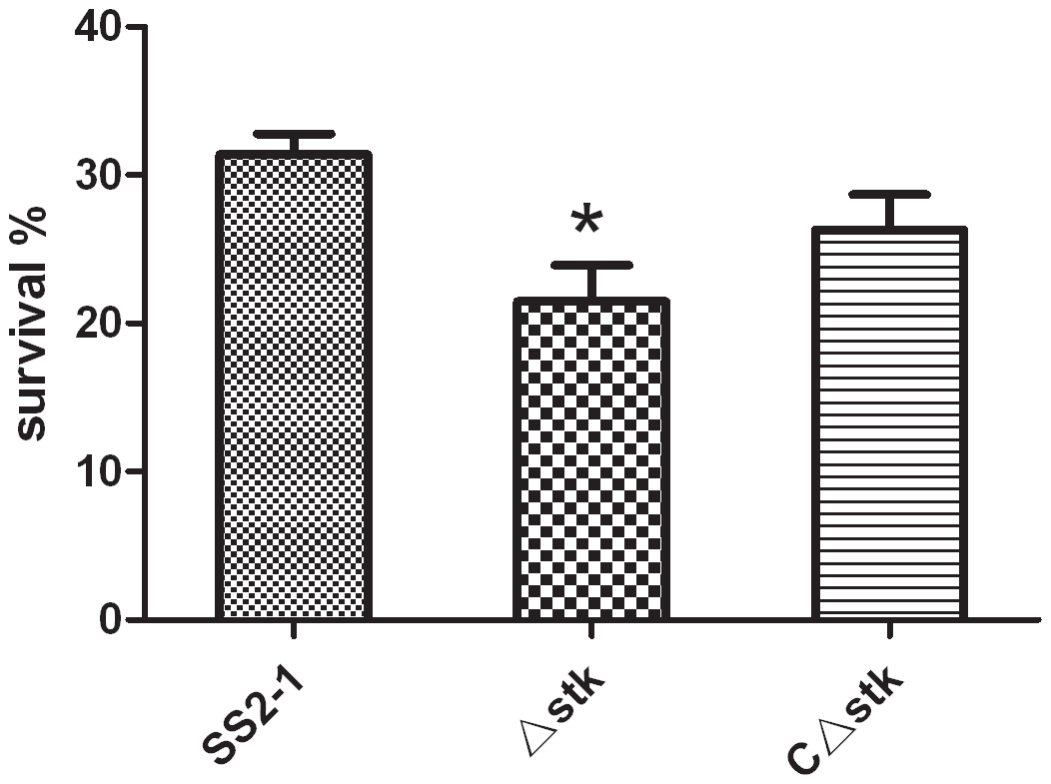
Survival of SS2-1 and Δ*stk* in pig whole blood. Mixtures were incubated at 37°C for 2 h. A value of 100% was given to the CFU at time 0 h. The percent survival rate of Δ*stk* significant reduced compared to SS2-1. (p = 0.0283).

Critical events in the development of disease are *S. suis* invasion from the mucosal surface into deeper tissues and the blood circulation. Therefore, *S. suis* survival in the blood is central to disease [36]. The impact of the *stk* mutation on the survival of SS2 in whole pig blood was tested. As shown in Fig.7, the survival rate of the wild-type strain SS2-1 was 31.2%, after 2 h incubation in whole blood. In contrast, the Δ*stk* was much more sensitive, with a survival rate of only 21.2% (p<0.05), and the survial ratio of the *C*Δ*stk* was 25.6%. Our results showed that *Ss*STK inactivation was significantly decreased the resistance of the pathogen to phagocytosis and killing in whole blood.

### The absence of *Ss*STK impairs *S. suis* virulence in CD1 mouse model

To study the effect of the *stk* mutation on virulence, we injected CD1 mice via the ip. route with either SS2-1, Δ*stk*, or *C*Δ*stk*. Mortality of mice was observed 7 days after the challenge. As shown in Table 3, the LD_50_ values were 2. 89×10^8^ CFU/mouse in Δ*stk*, 4.2×10^7^ CFU/mouse in SS2-1 and 1.53×10^8^ CFU/mouse in CΔ*stk*. The LD_50_ value of Δ*stk* was seven-fold higher than that of SS2-1. Therefore, the virulence of the Δ*stk* was lower than that of SS2-1 but could be restored in CΔ*stk*. The experimental infection in the mice strongly suggested that *Ss*STK played an important role in the pathogenesis of SS2.

**Table 3:** Calculations of LD_50_ on SS2-1 and its derivatives for CD1mice.

*Challenge dose (CFU/mouse)*	*Number of death/total*			*Death percent (%)*		
	SS2-1	Δ*stk*	*C*Δ*stk*	SS2-1	Δ*stk*	*C*Δ*stk*
2.0×10^9^	10/10	10/10	10/10	100	100	100
4.0×10^8^	10/10	6/10	8/10	100	60	80
8.0×10^7^	7/10	1/10	3/10	70	10	30
1.6×10^7^	2/10	0/10	0/10	20	0	0
3.2×10^6^	0/10	0/10	0/10	0	0	0
LD_50_	4.2×10^7^ CFU	2.89×10^8^ CFU	1.52×10^8^ CFU			

**Figure 8:**
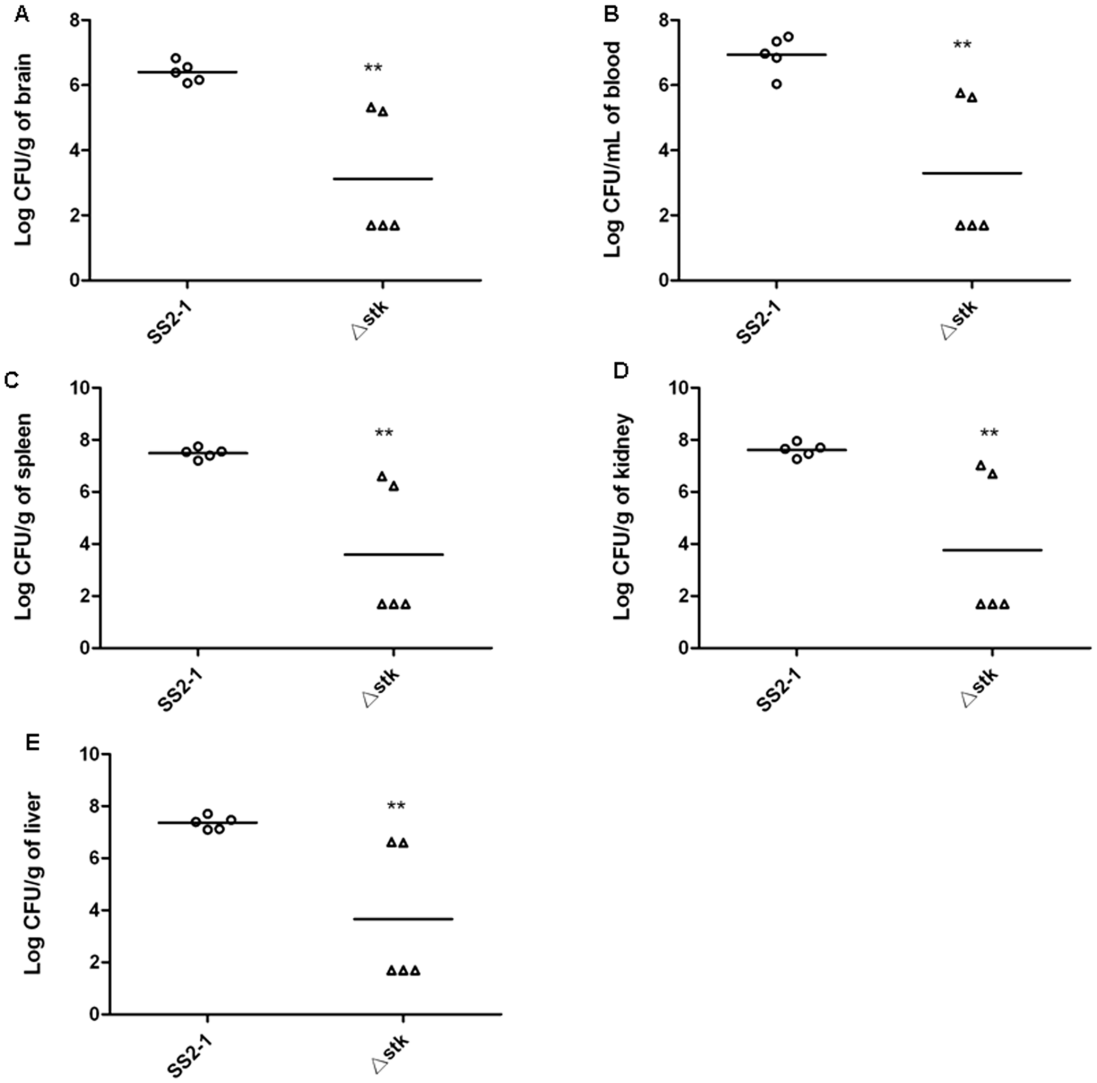
Bacterial viable in infected organs of mice (A:brain, B:blood, C:spleen, D:kidney, E:liver). Organ homogenates in which bacteria were not detected were arbitrarily assigned a value of 50 CFU, corresponding to the lower limit of the assay. Data were means ± SD of bacterial colonies from five mice.(**p<0.01).

To better evaluate the virulence attenuation of Δ*stk*, colonization experiments were carried out. Bacteria could be recovered from different organs (liver, spleen, kidney, brain and blood), which showed clinical symptoms of *S. suis* infection(shown in Table 4 and Fig 8). When mice were infected with Δ*stk* mutant strain, a distinct reduction of recovered bacterial numbers from different organs were observed compared to those mice infected with wild type stain (P<0.01). Organ homogenates in which bacteria were not detected were arbitrarily assigned a value of 50 CFU, corresponding to the lower limit of the assay [51]. The results of bacterial loads provided more evidence that *stk* inactivation severely impaired the virulence of SS2.

**Table 4:** Colonization analysis of Δ*stk* in various tissues of mice (×10^7^ CFU/g tissue).

	*Liver*	*Kidney*	*Brain*	*Spleen*	*Blood*
SS2-1	2.50±0.12	5.10±0.11	0.145±0.01	3.47±0.13	3.07±0.02
	1.25±0.03	2.92±0.14	0.245±0.03	2.49±0.12	2.2±0.05
	2.97±0.13	8.97±0.07	0.361±0.02	5.62±0.25	0.694±0.13
	5.09±0.08	4.52±0.15	0.679±0.05	3.56±0.08	0.93±0.04
	1.33±0.05	1.83±0.04	0.115±0.05	1.60±0.15	0.108±0.05
Δ*stk*	0.420±0.03	0.495±0.005	0.021±0.006	0.172±0.009	0.0425±0.006
	0.398±0.04	1.07±0.02	0.0157±0.008	0.402±0.012	0.0578±0.007
	-	-	-	-	-
	-	-	-	-	-
	-	-	-	-	-

Data are expressed as mean number of bacteria from three repeats.

-: No bacterial recovered from mice.

### The Δ*stk* mutant is attenuated in the piglet model of infection

**Figure 9:**
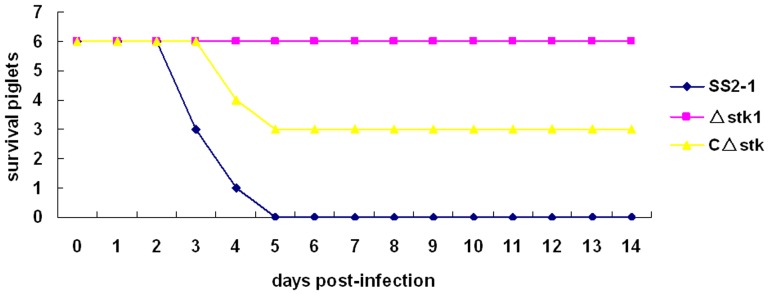
Survival curves for piglets in experiment infection. Six piglets in each group were intravenously inoculated with 5×10^6^ CFU/piglet of SS2-1, Δ*stk* and *CΔstk,* respectively. Two piglets were inoculated with PBS and served as a control.

To further delineate the role of *stk* in *S. suis* virulence, we conducted a trial in pigs, which are the natural hosts of infection. Piglet infection experiments showed that all of the piglets infected with SS2-1 at the dose of 5×10^6^ CFU/piglet developed hyperthermia, depression, lameness, and swollen joints during the first 48 h. Later, most of the typical symptoms, including shivering, central nervous system failure and respiratory failure were observed, and all piglets died within 5 days PI. In contrast, all six piglets infected with the Δ*stk* survived and did not develop serious symptoms throughout the experiment. In the CΔ*stk* infected group, three of six piglets presented sever clinical symptoms, and died from day 3 to day 5 PI. All survived animals were euthanased at day 14 PI. The experimental infection on piglets also indicated that SsSTK contributed significantly to the virulence of SS2(shown in Fig 9).

### Transcriptional analysis of virulence genes in SS2 strains

**Figure 10:**
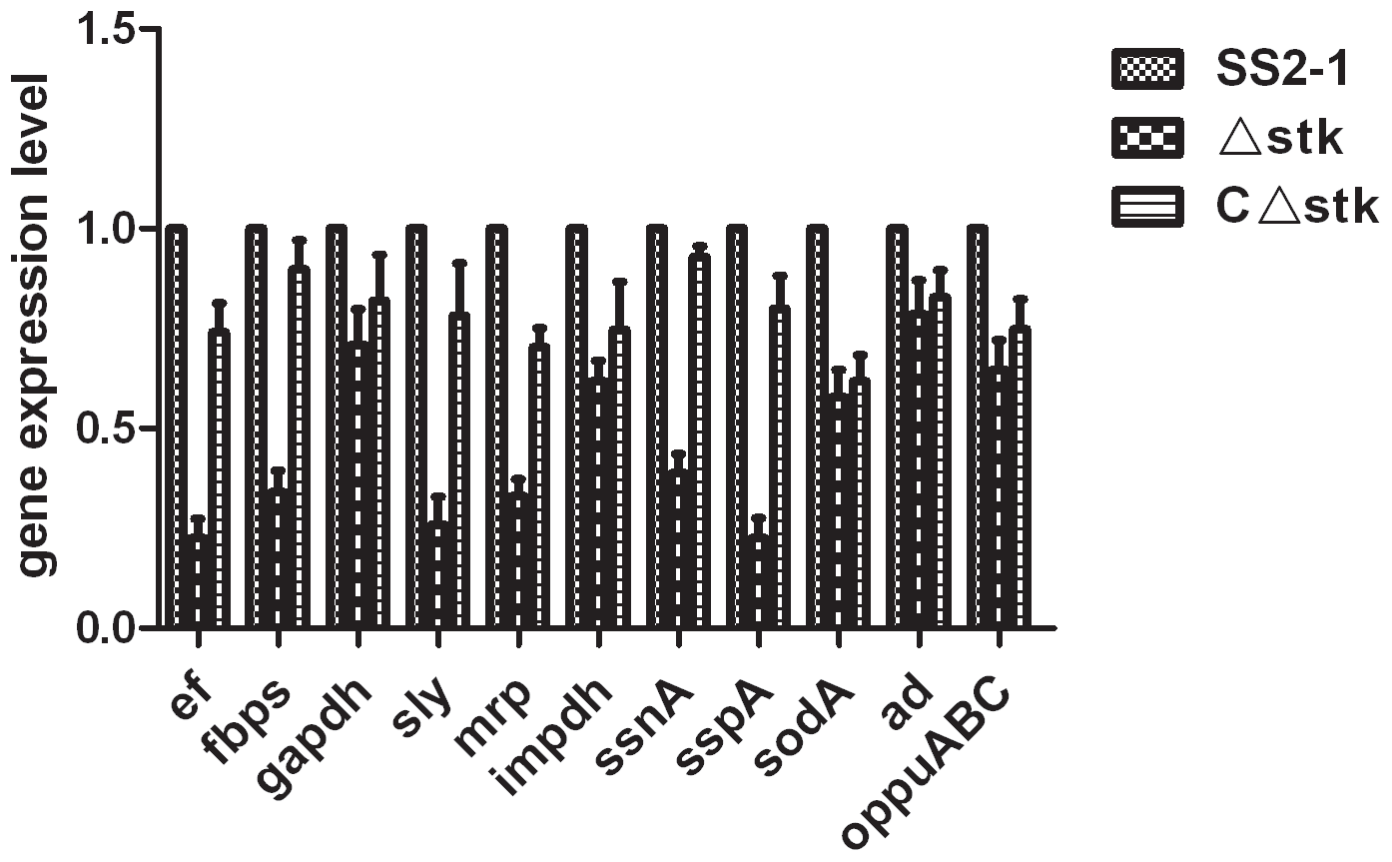
Virulence gene expression of different strains *in vitro*. Total RNA was extracted from SS2-1, Δ*stk* and *C*Δ*stk* grown in THY medium at an OD600 of 0.6–0.8 and used for qRT-PCR analysis. The mRNA level of each gene was normalized to that of *16S rRNA*. Results are shown as relative expression ratios compared to expression in the parental strain SS2-1. Data from three independent assays are presented as the means±SE. Differences between SS2-1 and Δ*stk* was determined by two-way ANOVA. (**p<0.01).

Previous results suggested a significant role for *Ss*STK in the pathogenicity of SS2, as it is in other *Streptococci* [5]–[8]. Several microbial determinants, such as Fbps and Gapdh, contribute to adherence and colonization [29], [52], SodA, AD and oppuABC, involved in various stress response [50], [53], [54]. Thus, the expression profiling of virulence factors association with adherence, colonization, stress response and other well-known virulent genes was analyzed by qRT-PCR with the three strains *in vitro*. As shown in Fig.10, the expression level of the stress response genes *sodA, ad* and *oppuABC* was decreased by 22–43%, compared with the parental strain SS2-1 (P<0.01). In C*Δstk,* the expression of these genes (*sodA, ad* and *oppuABC*) was reverted and higher than Δ*stk*, but there was no significant difference between Δ*stk* and CΔ*stk* (P>0.05). The expression level of adhesin genes *gapdh* and *fbps* was decreased by 30–66%. There were significant differences in *gapdh* and *fbps* transcription between the SS2-1and Δ*stk* (p<0.001). In *C*Δ*stk*, the expression level of *gapdh* and *fbps* were up to 82%, 89.8% of SS2-1, respectively. The expression level of other virulent genes such as *mrp, ef*, *impdh, sly, sspA* and *ssnA* of Δ*stk* was decreased to 0.33, 0.23, 0.62, 0.26, 0.39,and 0.23, respectively, as compared with the parental strain SS2-1 (P<0.01). The expression levels of these virulence factors were restored in the complemented strain *C*Δ*stk*.

## Discussion

Bioinformatics analysis revealed that a homologue of the serine/threonine kinase StkP of *S. pneumoniae* is highly conserved in *S. suis*. Eukaryotic-like signaling molecule StkP in *S. pneumoniae* has been studied extensively and found to regulate pleiotropic functions that include virulence, competence, antibiotic resistance, growth and stress response of the pathogen [7], [15], [16], [55]. In addition, StkP was a global regultor that influenced the transcription of approximately 4% of genome that includes genes involved in cell wall biosynthesis, oxidative stress, DNA repair, iron uptake and metabolism. Consistent with these observations, StkP mutants showed increased sensitivity to acid pH, temperature, oxidative and osmotic stress [15]. In this study, we created an ESTK mutant Δ*stk* in SS2-1, with an aim to investigate the function of the homologous ESTK in the pathogenesis of SS2.

Like some other *Streptococci* [5]–[8], the *stk* gene is adjacent to, and as we show here co-transcribed with, the *stp* gene encoding the cognate phosphatase. Deletion of *stk* did not have an effect on the transcription of *stp*, as shown by quantitative RT-PCR (data not shown). The Δ*stk* did not display significant changes in growth properties under nonstress conditions as in other *Streptococci* [6], [8], [15]. Light microscopic observations revealed that the *stk* mutant cells connected to each other to form a long cell chains compared with those formed by the parental strain (Fig. 5A),as has been reported for *S. agalactiae* (GBS) Stk1 deletion or both Stk1/Stp1 (double mutant) strains [6]. Most *stk* mutant cells sedimented when grown overnight, while the broth culture of wild-type cells was turbid, with fewer sedimented cells (Fig. 5B). The similar observations was also reported in *S. pyogenes* (GAS) SP-STK mutants [5].As the growth rate of the cells was not affected in the *stk* mutant, the difference in sedimented cells could be attributed to its longer cell chain length. The results suggested that SsSTK involved in the bacterial growth and morphology.

It has been suggested that diseases caused by *S. suis* begin with colonization of the nasopharyngeal tissue and that the interaction of *S. suis* with respiratory tract epithelial cells is central to the initiation of the disease process [56]. We compared the parental strain and mutant strain Δ*stk* and found a significant decrease in the mutant strain's adhesion to HEp-2 cells. Similar results has been reported in GAS for SP-STK mutants [5]. This observation was further confirmed by qRT-PCR analysis in which the expression level of adhesin genes (*fbps* and *gapdh*) of Δ*stk* were significantly decreased (Fig.10). Previous findings have suggested that adhesins may contribute to the pathogenicity of *S. suis* by mediating bacterial adherence [29], [52].Our results indicated that SsSTK mediates cell adhesion and virulence via controlling the expressions of *fbps* and *gapdh*.

To induce disease, *S. suis* must be able to survive in the bloodstream after its transmission via the respiratory tract [23]. We also compared the survival of the mutant and wild-type strains in whole pig blood. The result showed that the wild-type strain was much more resistant than the mutant strain. Similar results have been reported for SS2 for mutants in a subtilisin-like proteinase [44]. In *S*. *agalactiae*, the *stk1* expression also has shown to be important for survival of GBS in whole blood [38]. Previous investigations have shown that STK contributes to colonization and bacterial persistence during infections, such as the PrkC of *E. faecalis* [10] and the StkP of *S. pneumoniae* [7].The results of our *in vivo* colonization experiments showed that the Δ*stk* displayed significantly reduced bacterial colonization in tissues, including the liver, kidney, spleen, brain, and blood. This suggests that the absence of *stk* might lead to fewer bacteria *in vivo* and cause less tissue damage to the host post infection.

During the development of disease, *S. suis* strains have to invade deeper tissues and reach the blood circulation. Consequently, they have to adapt to an array of adverse environmental conditions such as elevated temperature, different pH, increased osmolality and oxygen pressure. Due to the transmembrane topology of STK and the presence of an extracellular sensor domain containing reiterated PASTA (penicillin-binding protein and serine/threonine kinase-associated) signature sequences [10], [57], we hypothesized that SsSTK, containing four repeated PASTA domains, could transmit environmental cues into the cell, as it is in other *Streptococci* [15], [18], [54]. Therefore, we investigated the growth characteristics of Δ*stk* mutant under different stress conditions. The results demonstrated that the Δ*stk* mutants showed defects in their ability to grow at various stress conditions, such as high temperature, low pH, oxidative stress and high osmolarity, compared with the wild-type strain. Similar results have been reported for SS2 for trigger factor mutants [58].The significantly decreased stress tolerance pattern of Δ*stk* mutant strain may be due to the down-regulation of some stress response genes *sodA, ad* and *oppuABC*(decreased by 22%–43%)(Fig.10). It has been demonstrated that the SodA and AD were involved in oxidative stress and acidic pH stress in *S. suis*, respectively [50], [53]. In GAS, *oppuABC* encodes a glycine betaine/proline transport protein that can protect bacterial cells from osmotic shrinkage in the presence of high salt concentrations [54], [59]. The lower tolerance of the Δ*stk* mutant strain to various environmental stresses might be a major contributor to the attenuated virulence of bacterial, since such mutant cells would be less likely to survive in the host.

*In vivo* studies with animal models (mice or rats) of infection have shown that mutant strains defective in STK expression, including *stk1* of *S*. *agalactiae* [6], *Stk* of *S. pyogenes* [54], *Stk1* of *S. aureus* [22] and *StkP* of *S. pneumoniae* [7],are less virulent than the wild-type strains. To evaluate the effect of *stk* inactivation on virulence in SS2, CD1 mice and piglets were experimentally infected with the bacteria. In CD1 mice, the LD_50_ value of Δ*stk* was significantly increased (seven-fold higher) compared with that of the wild-type strain, whereas the virulence was restored in the complemented strain. In the piglets model, the Δ*stk* also has been shown to significantly lower lethality than the wild-type strain(Fig.9). Together, these results indicated that deletion of *stk* resulted in the attenuated virulence of SS2. The significantly decreased virulence of STK-deficient strain can be attribute to the down-regulation of several known and putative virulence genes such as *mrp, ef*, *fbps*, *gapdh*, *impdh, sly, sspA*, *ssnA*, *sodA*, *ad* and *oppuABC*, involving in the adherence, colonization, stress response and virulence of *S. suis* (Fig.10). qRT-PCR analysis also showed that when *stk* gene was reverted, the virulence of SS2 was reinforced, which supported the results of adherence to HEp-2 cell, survival in swine whole blood and various stress conditions, and experimental infection models.

In conclusion, we have performed a functional genetic description of an orthologous ESTK in SS2 and revealed new insights into the requirement for *stk* in the pathogenesis of SS2 infection. Our results strongly suggested that the SsSTK may coordinate and regulate some important factors involved in various steps of bacterial pathogenesis, including adherence to host cell, survival in various stress environments and colonization in host tissues. Further investigations are necessary to be conducted to define the genes involved in signal transduction of SsSTK, which will provide more in-depth insights into the ESTK-associated regulatory network and pathogenesis in SS2.
